# Identifying Tau proteopathic species isolated from human brains with Alzheimer’s disease

**DOI:** 10.1002/alz.092798

**Published:** 2025-01-03

**Authors:** Noé Quittot, Dhanush Sivasankaran, Victoria Derosla, Anne Wiedmer, Romain Perbet, Bradley T. T Hyman

**Affiliations:** ^1^ Massachusetts General Hospital, Harvard Medical School, Boston, MA USA

## Abstract

**Background:**

In Alzheimer’s disease (AD), the spread of Tau proteopathic seeds across the cerebral cortex parallels the disease progression. Previously, it was shown that isolating high‐molecular‐weight (HMW) Tau species via size exclusion chromatography (SEC) from human brain lysate of AD patients resulted in the enrichment of Tau aggregation‐prone species. However, whether the HMW Tau population contain a homogenous or heterogeneous mixture of Tau species is still unknown. Therefore, this research proposal aims at fractionating the HMW Tau population to identify Tau proteopathic seeds.

**Method:**

Human brains tissue from 9 AD cases have been homogenized in PBS and the resulting lysates were then run through a SEC to isolate the HMW Tau. The HMW Tau population was then fractionated according to net charge of the Tau species by anion exchange chromatography (AIEX). Following each chromatographic strategy step, the bioactivity of the different fractions was assessed via a cell‐based assay to track the aggregation‐prone Tau species.

**Result:**

First, we have demonstrated that these chromatographic strategies can be carried out under near‐physiologic pH and salt conditions, maintaining Tau proteopathic seeds bioactivity and hence their bioactive conformation. Second, we confirmed the presence of HMW Tau subspecies, and more importantly both non‐bioactive and bioactive HMW Tau species that co‐elute from the size column. Finally, the AIEX separation technique revealed that the bioactive species were eluted later than the non‐bioactive, suggesting that the bioactive species are more negatively charged and more heavily post‐translationally modified.

**Conclusion:**

The HMW Tau population contains various subspecies with both non‐bioactive and bioactive species, that can be separated according to their charge and their retention time on an AIEX column. In sum, these results suggest that the HMW fraction purified by SEC is a mixture of subspecies, and that the overall net charge of these subspecies appears to be a crucial biochemical feature correlating with HMW Tau bioactivity.

